# Dissociation of structural and functional connectomic coherence in glioma patients

**DOI:** 10.1038/s41598-021-95932-5

**Published:** 2021-08-18

**Authors:** Kerstin Jütten, Leon Weninger, Verena Mainz, Siegfried Gauggel, Ferdinand Binkofski, Martin Wiesmann, Dorit Merhof, Hans Clusmann, Chuh-Hyoun Na

**Affiliations:** 1 Department of Neurosurgery, RWTH Aachen University, Pauwelsstraße 30, 52074 Aachen, Germany; 2 Imaging and Computer Vision, RWTH Aachen University, Templergraben 55, 52074 Aachen, Germany; 3 Institute of Medical Psychology and Medical Sociology, RWTH Aachen University, Pauwelsstraße 19, 52074 Aachen, Germany; 4 Division of Clinical Cognitive Sciences, RWTH Aachen University, Pauwelsstraße 17, 52074 Aachen, Germany; 5 Department of Diagnostic and Interventional Neuroradiology, RWTH Aachen University, Pauwelsstraße 30, 52074 Aachen, Germany; 6 Center for Integrated Oncology Aachen Bonn Cologne Duesseldorf (CIO ABCD), Aachen, Germany

**Keywords:** Head and neck cancer, Tumour heterogeneity

## Abstract

With diffuse infiltrative glioma being increasingly recognized as a systemic brain disorder, the macroscopically apparent tumor lesion is suggested to impact on cerebral functional and structural integrity beyond the apparent lesion site. We investigated resting-state functional connectivity (FC) and diffusion-MRI-based structural connectivity (SC) (comprising edge-weight (EW) and fractional anisotropy (FA)) in isodehydrogenase mutated (IDHmut) and wildtype (IDHwt) patients and healthy controls. SC and FC were determined for whole-brain and the Default-Mode Network (DMN), mean intra- and interhemispheric SC and FC were compared across groups, and partial correlations were analyzed intra- and intermodally. With interhemispheric EW being reduced in both patient groups, IDHwt patients showed FA decreases in the ipsi- and contralesional hemisphere, whereas IDHmut patients revealed FA increases in the contralesional hemisphere. Healthy controls showed strong intramodal connectivity, each within the structural and functional connectome. Patients however showed a loss in structural and reductions in functional connectomic coherence, which appeared to be more pronounced in IDHwt glioma patients. Findings suggest a relative dissociation of structural and functional connectomic coherence in glioma patients at the time of diagnosis, with more structural connectomic aberrations being encountered in IDHwt glioma patients. Connectomic profiling may aid in phenotyping and monitoring prognostically differing tumor types.

## Introduction

With the increasing awareness of a network-based rather than traditional localizationist understanding of the organization of cerebral functions^1,2^, determining the systemic, i.e. whole-brain, impact of chronically progressive brain lesions such as in glioma patients seems increasingly relevant^3–7^. While tumor lesions may not obviously correlate with the functional (e.g. cognitive or affective) impairment in these patients, it is understood that by tumor-induced disruptions of cortico-cortical connections, not only perilesional brain regions, but distant brain areas can be functionally affected as well^7–9^. Moreover, infiltrative glioma cells are known to invade macroscopically unaffected brain regions by occult migration along white matter (WM) bundles^10,11^, and microstructural WM alterations have been reported in diffuse glioma even in the normal-appearing WM, distant to the primary lesion site^12–14^.

Diffusion-weighted imaging (dMRI) provides estimates of macroanatomic WM tracts, as well as measures for microstructural characterization of WM properties in vivo. dMRI is used for surgical treatment planning by tracking peritumoral eloquent WM tracts, or has been applied to estimate the extent of diffuse tumor infiltration^15–17^. dMRI can furthermore be used for mapping whole-brain WM structural connectivity (SC), based on whole-brain parcellation with diffusion based tractography algorithms, estimating the number of all streamlines connecting each parcellated region with one another^18^. Thereby, an individual WM structural connectome can be delineated in a connectivity matrix, depicting intra- as well as interhemispheric measures of diffusion based SC. Although glioma is increasingly acknowledged as a whole-brain disease, diffusion-based analyses of tumor-related WM alterations have so far mainly been confined to selected regions or volumes of interest^13–15,19^, while network-specific SC analyses or the whole-brain structural connectome have as yet only scarcely been addressed in tumor patients^3,5,20^.

Information on functional connectivity (FC) can be derived from functional MRI based on spatiotemporally correlated blood-oxygen-level-dependent (BOLD) signal fluctuations, and has frequently been used to investigate functional network representations in tumor patients^9,21–23^. Under consideration of cognitive and/or sensorimotor impairment in patients, in particular task-independent (resting-state) functional MRI has evolved as of increasing interest for potential applications in the clinical setting. Moreover, imaging acquisition times in resting-state functional MRI (rs-fMRI) are patient-friendly, as FC can be assessed with regard to various different networks based on a single imaging data acquisition. Previous studies have frequently shown correlations of functional impairment with network-specific alterations in rs-fMRI connectivity in glioma patients^21,23,24^. Findings did however not always seem conclusive, as increases as well as decreases in network-specific FC have been reported to be associated with functional impairment in patients^9,23,25^, and the relation to underlying structural alterations remains unclear. Glioma-associated aberrations in whole-brain FC have recently been shown to be associated with tumor grade and isodehydrogenase (IDH) mutation status, with stronger abnormalities described in the prognostically less favorable IDH-wildtype (IDHwt) patient group^26^.

How these FC aberrations relate to SC alterations remains however elusive, as to date, only few studies investigated both SC as well as FC intraindividually in tumor patients^20^. As a major goal in surgical therapy is to maximally remove tumor tissue without evoking functional deficits, understanding the relation of structural and functional connectivity in tumor patients is highly relevant. With slowly destructive tumor growth being more likely to be paralleled by neuroplasticity, anatomical boundaries for eloquent brain regions might be significantly altered in glioma patients, increasing the risk of functional deficits in tumor surgery. Therefore, a better understanding of SC and FC alterations in glioma patients is of importance, as it could improve both neurooncological and functional outcome in surgical tumor therapy.

In how far SC and FC closely interrelate in glioma patients, whether they diverge regionally (e.g. depending on tumor site or -volume), depend on tumor growth dynamics (as indicated by the molecular genetic profile such as IDH-mutation status), or are impacted by neuroplasticity, has yet to be determined, and could furthermore aid in phenotyping prognostically differing tumor types.

We therefore sought to investigate both the structural and functional connectome in glioma patients compared to healthy controls, using diffusion-MRI and rs-fMRI. We chose a whole-brain approach in order to investigate global alterations of FC and SC. As connectivity however strongly differs in hub and non-hub regions, and as glioma associated FC changes have previously been suggested to be altered specifically with regard to hub and non-hub connectivity^5^, we additionally analyzed connectivity of the Default-Mode Network (DMN), as it comprises some of the core hub regions. We wanted to investigate, whether IDHmut and IDHwt glioma patients differ in whole brain and/or more specifically in hub connectivity.

Thus, whole-brain as well as DMN SC and FC were compared between patient groups and controls, and partial correlations were analyzed intermodally (SC/FC) and intramodally (intra-/interhemispherically) for the structural and functional connectome. We further aimed at analyzing intramodal correlations as an indicator of connectomic coherence, as it may relate to the global integration of long-range information processing. Please note that with ‘modality’ we refer to different measures of FC and SC (namely FC, EW and FA) and not categorically to either diffusion or functional MRI.

We expected alterations of the structural connectome in glioma patients to be associated with, albeit not necessarily spatiotemporally congruent, alterations of the functional connectome. We further hypothesized that IDHmut and IDHwt glioma patients would show differing patterns of connectomic alterations, with stronger disintegrity of the structural and functional connectome expected to be associated with the more invasive IDHwt glioma tumor type.

## Methods

### Participants

29 patients with cerebral glioma (mean age: 50 ± 17 years, 17 males, 28 right-handed, 19 LH PAT, 15 IDHmut) and 27 healthy controls (mean age: 46 ± 14 years, 17 males, 26 right-handed) were included in the study. They were part of a bigger sample of 36 patients and 30 controls, who were prospectively enrolled at a single university hospital center and were examined in detail in previous studies^25,27^. Only unilateral and histopathologically proven gliomas were finally included in the analysis. For further analyses, patients were split into subgroups according to their IDH-mutation status, resulting in patient groups of 15 IDHmut and (mean age: 37 ± 11 years, 11 males, 14 right-handed, 10 LH PAT) and 14 IDHwt patients (mean age: 65 ± 8 years, 6 males, 14 right-handed, 9 LH PAT). Histopathological diagnoses were determined according to the revised WHO tumor classification of 2016^28^, integrating histoanatomical and molecular genetic criteria, and IDH-mutation status was determined.

**Figure 1:**
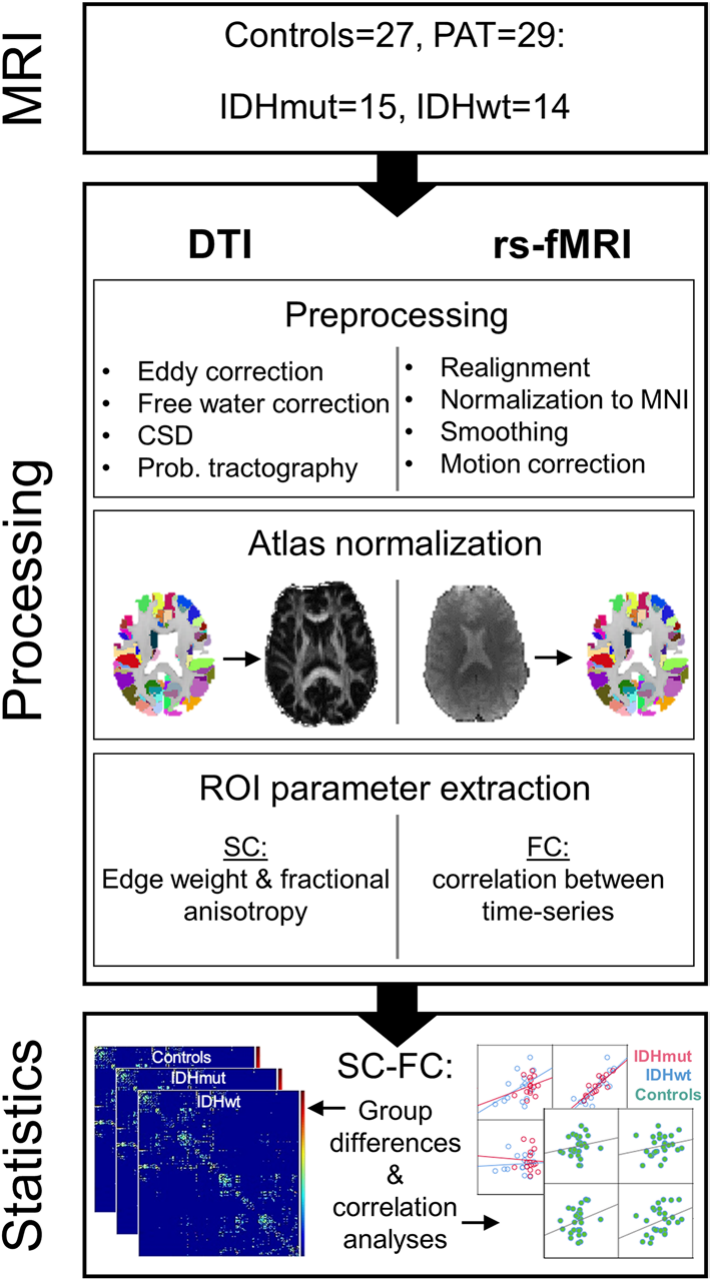
Flowchart of applied methods. The MRI examination was acquired preoperatively from 29 patients (PAT), of which 15 had isodehydrogenase (IDH) mutated, and 14 had wildtype gliomas. After tumor segmentation, diffusion tensor images (DTI) and resting-state functional MRI (rs-fMRI) data were preprocessed, and Brainnetome Atlas-based regions of interest (ROI) were extracted. Within these ROIs, the structural and functional connectivity (SC and FC) was computed to analyze between-group connectomic differences, as well as intra- and intermodal correlations for each group.

Only patients ≥ 18 and < 80 years of age with unilateral supratentorial tumors and a Karnofsky index of ≥ 70 were included in the study. All patients except two were naíve to tumor-specific treatment prior to enrollment in the study. Patients’ demographics and tumor characteristics can be found in Supplement 1. All participants gave informed written consent. The study was approved by the local ethics committee of the Medical Faculty of the University of the RWTH Aachen (EK294-15) and conducted in accordance with the standards of Good Clinical Practice and the Declaration of Helsinki. An overview of included subjects and all methods applied can be found in Fig. 1.

### MRI data acquisition

MRI examination was applied using a 3T Siemens Prisma MRI scanner equipped with a standard 20-channel head coil. The detailed scanning protocol is described in previous studies^25,27^, and comprised the following pulse sequences: First, a sagittal 3D T1 magnetization-prepared rapid acquisition gradient echo (MPRAGE) sequence was acquired [repetition time (TR) = 2300 ms, echo time (TE) = 2.01 ms, 176 slices with a slice thickness of 1 mm, flip angle = 9°, field of view (FoV) = 256 mm, voxel size = 1 mm isotropic, and 256 × 256 matrix]. For tumor identification purposes, a contrast-enhanced, T1-weighted turbo inversion recovery magnitude (TIRM) dark-fluid sequence was acquired (TR = 2200 ms, TE = 32 ms, slice thickness = 3 mm, flip angle = 150°, FOV = 230 mm, voxel size = 0.9 × 0.9 × 3.0 mm3, matrix = 256 × 256) as well as a T2-weighted TIRM dark-fluid scan were applied (TR = 9000 ms, TE = 79 ms, slice thickness = 3 mm, flip angle = 150°, FOV = 230 mm, voxel size = 0.9 × 0.9 × 3 mm3, matrix = 256 × 256). In addition, a fluid attenuation inversion recovery (FLAIR) sequence was applied (TR = 4800 ms, TE = 304.0 ms, number of slices = 160 with 1 mm slice thickness, FoV m s250 mm, and 1 mm isotropic voxel resolution), and RS-fMRI was implemented using echo planar imaging (EPI), including 300 whole brain functional volumes, TR = 2200 ms, TE = 30 ms, number of slices = 36 with 3.1 mm slice thickness, flip angle = 90°, and FoV = 200 mm.

### Preprocessing

Diffusion acquisitions were corrected for susceptibility-induced correction with FSL TOPUP as described in^29^, and for eddy currents and motion artifacts with FSL EDDY^30^. In five cases, in which the reverse-phase encoded image was corrupted, only FSL EDDY was applied. The T1 image, excluding the tumor, was segmented into WM, gray matter and CSF using FSL FAST. The tissue segmentation, as well as the Brainnetome parcellation^18^ were transformed into diffusion space using s symmetric diffeomorphic image registration of T1 image and diffusion acquisition^31^. To distinguish between diffusion anisotropy loss due to fiber degradation and signal loss caused by free water compartments, a free water correction was applied as described elsewhere^32,33^.

Probabilistic diffusion tractography was performed in native diffusion space using anatomical constraints of the transformed tissue segmentation map. Fiber orientations were obtained with constrained spherical deconvolution^34^. Using the obtained fiber orientation distribution function, probabilistic tractography, as implemented in Dipy^35^ was performed with a step size of 0.5 mm and a maximum angle between subsequent steps of 30°. Tracking seed point were set to the boundary between gray matter and WM using 3 × 3 × 3 seed points per voxel. Tracking was terminated if the FA value was below 0.15 or when the WM boundary was reached. All streamlines that did not terminate in gray matter or where the final length was less than 2 mm were discarded. The remaining streamlines were partitioned into fiber tracts depending on the gray matter start- and end regions as defined in the Brainnetome Atlas^18^.

Functional preprocessing was performed using SPM12^36,37^ as implemented in Matlab 9.5^38^. A detailed description of the image preprocessing protocol can be found in previous studies^25,27^. In brief, tumor lesions were segmented semi-automatically using the ITK-SNAP software version 3.4.0^39^ (http://www.itksnap.org/pmwiki/pmwiki.php?n=Downloads.SNAP3) and included perifocal T1 hypo- and T2-FLAIR hyperintensities for gliomas grade II-III, as well as T1 hypointensities and contrast-enhancing tumor for glioblastomas. Then, functional images were realigned to the mean functional volume, unwarped and coregistrated to the structural image. Structural and functional images were normalized (including a binary tumor mask in case of patients’ data), and functional images were smoothed with a 5 mm FWHM Gaussian kernel. Then, functional images were slice-time corrected and movement-related time series were regressed out with ICA-AROMA^40^. Data were high-pass filtered (> 0.01 Hz) and parcellated into a set of 246 predefined anatomical brain regions using the Brainnetome Atlas. A list of included left- and right-hemispheric brain regions can be found here (https://atlas.brainnetome.org/download.html).

### Whole-brain and DMN SC and FC analyses

**Figure 2:**
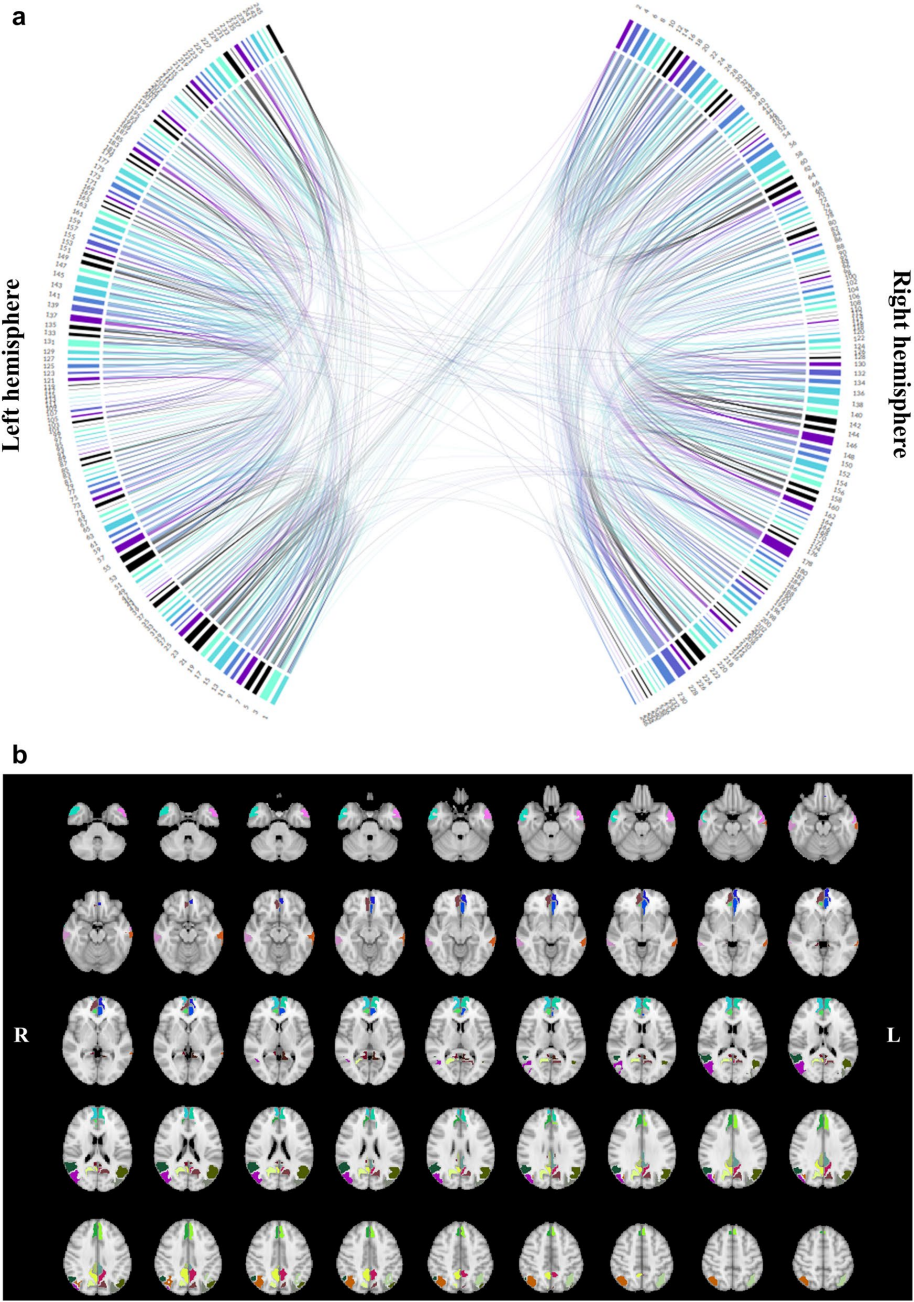
Regions of interest included in whole-brain and Default-Mode Network analyses. Investigated regions of interest (ROIs) were generated from Brainnetome Atlas parcellation, a list of brain regions can be found in Supplement 3 and on the Brainnetome homepage (https://atlas.brainnetome.org/download.html). (**a**) Of these 246 brain regions of the Brainnetome Atlas, only those ROIs that were interconnected by at least one streamline in all subjects of the control group were included in whole-brain analyses. Even numbers refer to right-hemispheric ROIs, whereas uneven numbers represent left-hemispheric ROIs. (**b**) For the analyses of the Default-Mode Network (DMN), atlas structures comprising hub regions such as the anterior cingulate cortex, posterior cingulate cortex, medial temporal gyrus and inferior parietal lobule were chosen.

To investigate differences in whole-brain distant (contralesional), local (ipsilesional) and interhemispheric SC and FC between patient groups and controls, individual subjects’ time-courses were extracted from parcellated atlas regions, which were used as ROIs. For quantification of SC, the EW between all pairs of ROIs was determined, resulting in a 246 × 246 SC matrix. EW was defined as the number of fiber connections between the two ROIs divided by the mean number of fibers originating or ending within these two ROIs. In addition, mean FA values were computed for all fibers connecting each ROI with one another, also resulting in a 246 × 246 SC matrix. With regard to FC, all ROIs’ mean time-series were computed and cross-correlated within each subject, resulting in a 246 × 246 FC matrix. Correlations were Fisher z-transformed. Only those ROIs interconnected by at least one streamline in controls were considered for further analyses. This included 619 left-hemispheric, 637 right-hemispheric, and 92 interhemispheric ROI connections (Fig. 2a). Based on these remaining ROIs, contra- and ipsi-lesional, as well as interhemispheric means of SC measures (EW, FA) and FC were computed and analyzed for differences between patient groups and controls.

**Figure 3:**
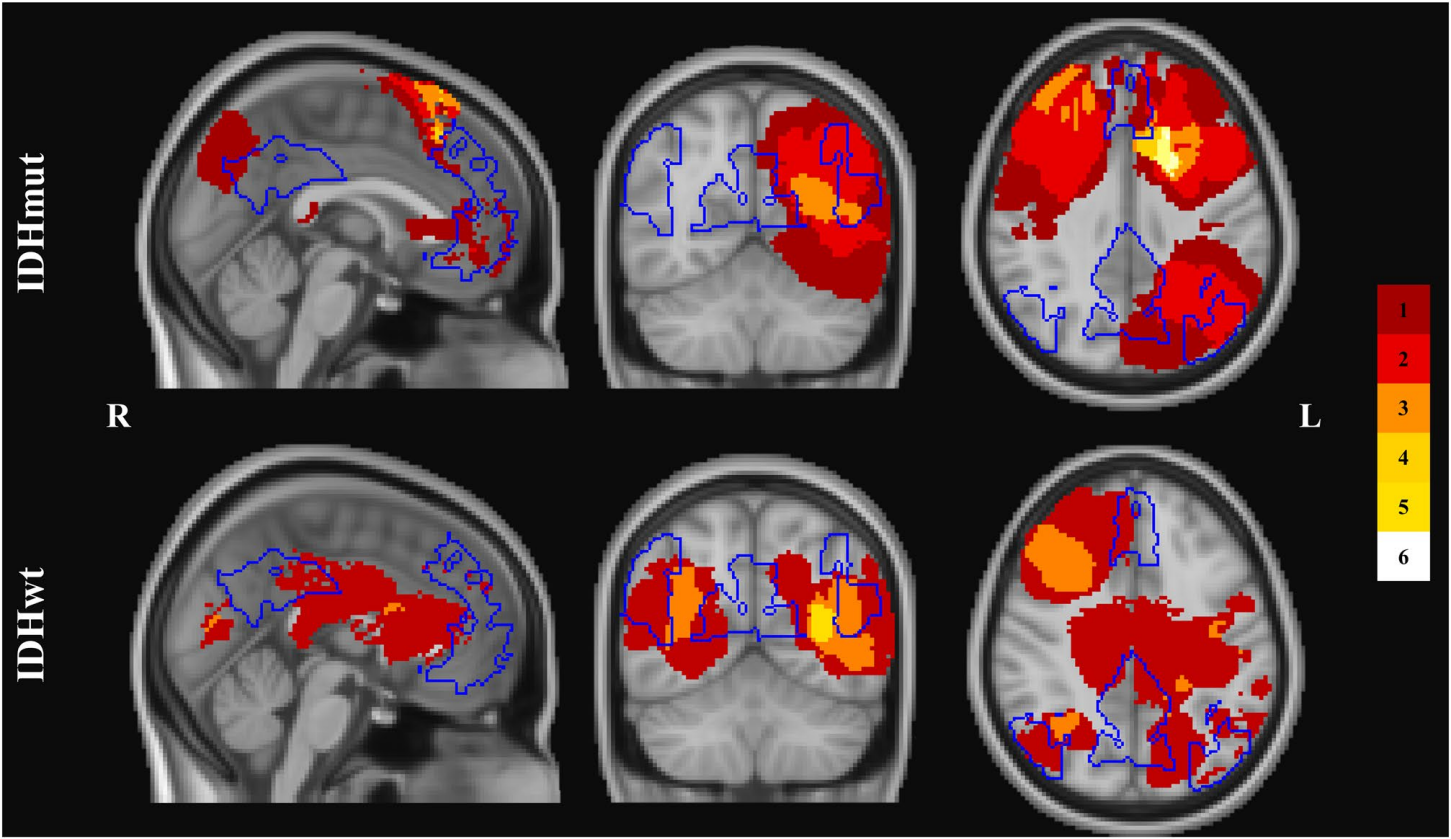
Overlap between Default-Mode Network regions and tumor locations. The distribution of right- and left- hemispheric tumor locations are displayed for isodehydrogenase-mutated (IDHmut) and wildtype (IDHwt) tumor patients. Regions with highest overlap in tumor occurrences are visualized in light yellow. Regions of the Default-Mode Network are overlaid in blue in order to visualize the overlap of patients’ tumor distribution and network representation.

To investigate DMN specific connectivity, Brainnetome Atlas structures comprising the anterior cingulate cortex, posterior cingulate cortex, medial temporal gyrus and inferior parietal lobule were chosen as ROIs and DMN-EW, DMN-FA and DMN-FC were computed (Fig. 2b). An overview of included DMN hub regions, overlaid on tumor locations of IDHmut and IDHwt can be found in Fig. 3. Within these DMN hub regions, mean contralesional, ipsilesional, and interhemispheric SC and FC were computed and compared across patient groups and controls.

### Statistics

All statistical analyses were performed with SPSS 24^41^. Data measures deviating more than 2.5 standard deviations (SD) from the group-specific mean were regarded as outliers and corrected for by being replaced by the “worst” group-specific score on that respective variable.

In healthy controls, the mean of left- and right-hemispheric SC and FC was computed and used as baseline (“healthy”) to compare contra- and ipsilesional connectivity between groups.

The gender ratio did not differ significantly between IDH groups and controls (Chi^2^ Tests χ^2^ = 2.851, *p* = 0.354).

### Between-group differences in whole-brain SC and FC

To investigate whole-brain contralesional, ipsilesional, and interhemispheric tumor-related effects on SC and FC, differences in EW, FA and FC between patient groups and controls were explored. Three multivariate ANOVAs were applied (for ipsi-, contralesional, and interhemispheric SC, FA and FC, respectively), including group (IDHmut, IDHwt, controls) as between-subject factor, hemispheric SC and FC (first: contralesional EW, FA and FC, second: ipsilesional EW, FA and FC, third: interhemispheric EW, FA and FC) as dependent variable.

### Between-group differences in DMN SC and -FC

Possible differences in DMN-EW, DMN-FA and DMN-FC between patient groups and controls were analyzed applying three multivariate ANOVAs, including group (IDHmut, IDHwt, controls) as between-subject factor, and hemispheric DMN-EW, DMN-FA and DMN-FC (first: contralesional DMN-EW, DMN-FA and DMN-FC, second: ipsilesional DMN-EW, DMN-FA and DMN-FC, third: interhemispheric DMN-EW, DMN-FA and -FC) as dependent variable.

### Intra- and intermodal partial correlations of whole-brain and DMN SC and FC

The relationship between EW, FA and FC (intramodal: EW-EW, FA-FA, FC-FC; intermodal: EW-FA, EW-FC, FA-FC) was analyzed for patient groups and controls separately using Pearson’s partial correlation analyses, controlling for effects of age. Correlation analyses were tested two-sided with a significance level of *p* < 0.05 and Bonferroni-corrected for multiple testing (contra-, ipsi-tumoral, and interhemispheric EW, FA and FC; adjusted *p* = 0.006).

All statistical comparisons were tested two-sided with a significance level of *p* < 0.05 and Bonferroni-corrected.

## Results

### Between-group differences in whole-brain SC and FC

The multivariate ANOVA of contralesional EW, FA and FC between patient groups and controls revealed significant group differences (*F*(6, 104) = 2.59, *p* = 0.022). Here, group differences were present in FA (*F*(2, 53) = 6.15, *p* = 0.004), whereas EW and FC did not reach significance. Post-hoc comparisons showed significantly higher FA values for IDHmut as compared to IDHwt patients (*p* = 0.008), and as compared to controls (*p* = 0.011).

Similarly, ipsilesional group differences reached significance (*F*(6, 104) = 3.09, *p* = 0.008). Here, too, group differences were found for FA only (*F*(2, 53) = 7.20, *p* = 0.002). Post-hoc comparisons revealed significantly lower FA values for IDHwt patients as compared to patients with IDH-mutation status (*p* = 0.002) and controls (*p* = 0.012).

**Figure 4:**
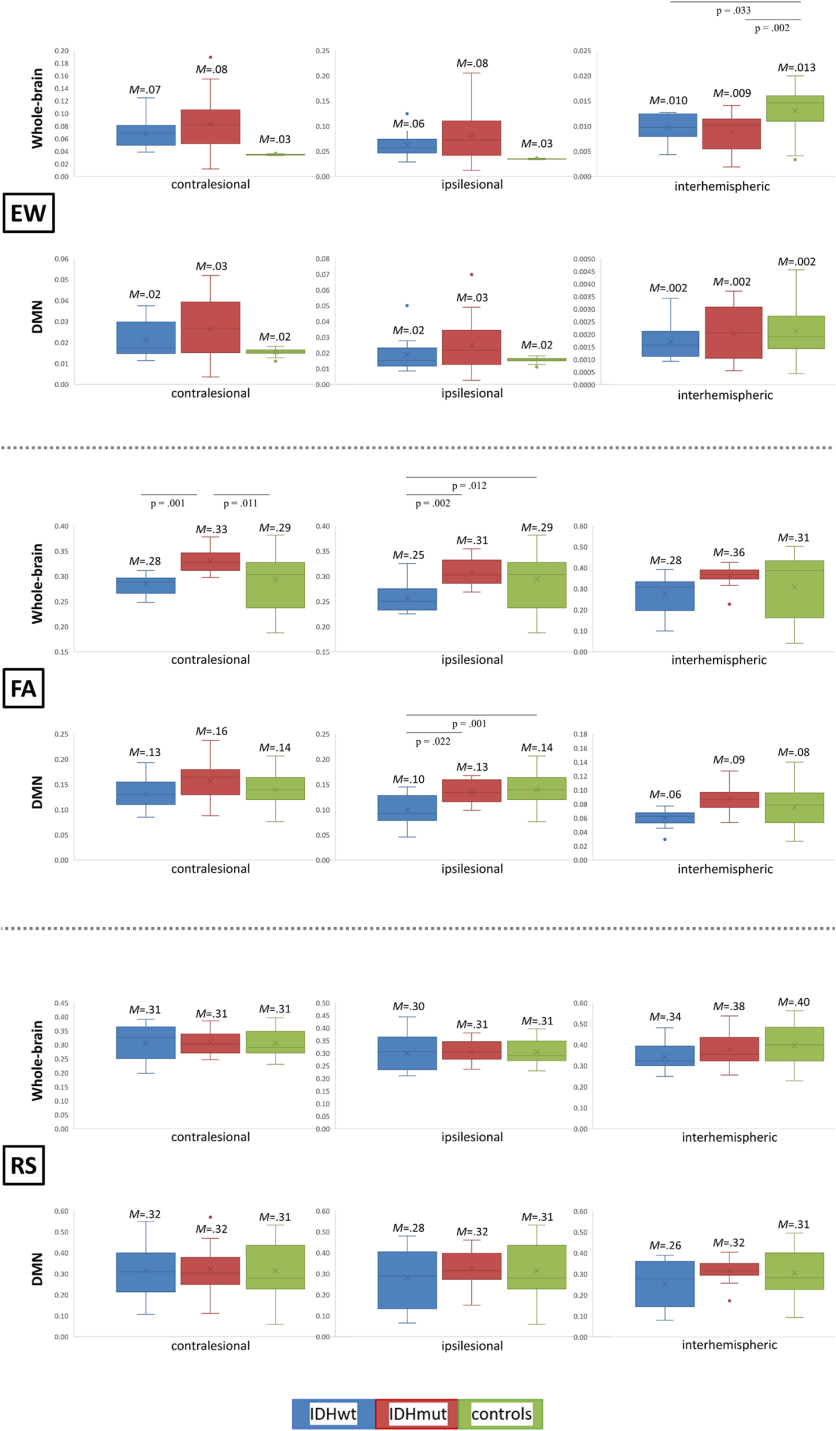
Group differences in structural and functional connectivity. Multivariate analyses of variance revealed group differences in edge weight (EW), fractional anisotropy (FA), and functional connectivity (FC) between isodehydrogenase-wildtype (IDHwt, blue), IDH-mutated gliomas (IDHwt, red), and healthy controls (green). Significances for each analysis were computed two-sided with a significance level of *p* < .05, including post-hoc Bonferroni-correction.

The multivariate ANOVA of interhemispheric EW, FA and FC revealed significant group differences (*F*(6, 104) = 3.87, *p* = 0.002), particularly regarding EW (*F*(2, 53) = 7.43, *p* = 0.001). Post-hoc analyses indicated higher EW values for healthy controls as compared to IDHmut (*p* = 0.002) and IDHwt patients (*p*.033). Multivariate analyses results are visualized in Fig. 4.

### Between-group differences in DMN SC and -FC

Significant group differences were found regarding ipsilesional SC (*F*(6, 104) = 3.01, *p* = 0.009). More specifically, group differences were present for FA (*F*(2, 53) = 7.26, *p* = 0.002). Post-hoc comparisons revealed significantly lower FA values for IDHwt patients as compared to IDHmut patients (*p* = 0.022) and controls (*p* = 0.001), see Fig. 4. The remaining multivariate ANOVAs did not reveal significant group differences, neither for contralesional nor interhemispheric DMN-EW, DMN-FA or DMN-FC.

### Intra- and intermodal partial correlations of whole-brain SC and FC

In healthy controls, partial correlation analyses revealed significant intramodal correlations. In particular, left-and right-hemispheric connectivity measures were positively correlated with each other and with interhemispheric connectivity in all modalities except for EW, in which only left- and right hemispheric EW were correlated (EW_LEFT_-EW_RIGHT_: *r*_partial_ = 0.53, *p* = 0.005; FA_LEFT_-FA_RIGHT_: *r*_partial_ = 0.91, *p* = 0.000; FA_LEFT_-FA_INTERHEM_: *r*_partial_ = 0.88, *p* = 0.000; FA_RIGHT_-FA_INTERHEM_: *r*_partial_ = 0.78, *p* = 0.000; FC_LEFT_-FC_RIGHT_: *r*_partial_ = 0.75, *p* = 0.000; FC_LEFT_-FC_INTERHEM_: *r*_partial_ = 0.85, *p* = 0.000; FC_RIGHT_-FC_INTERHEM_: *r*_partial_ = 0.72, *p* = 0.000).

In IDHmut patients, no intramodal correlations of SC measures were found, while intramodal correlations of FC remained significant (FC_CONTRA_-FC_IPSI_: *r*_partial_ = 0.85, *p* = 0.000; FC_CONTRA_-FC_INTERHEM_: *r*_partial_ = 0.80, *p* = 0.001; FC_IPSI_-FC_INTERHEM_: *r*_partial_ = 0.76, *p* = 0.002).

IDHwt patients showed no intramodal correlations of SC measures and even a further loss in intramodal correlation within the functional connectome (FC_CONTRA_-FC_IPSI_: *r*_partial_ = 0.80, *p* = 0.001).

**Figure 5:**
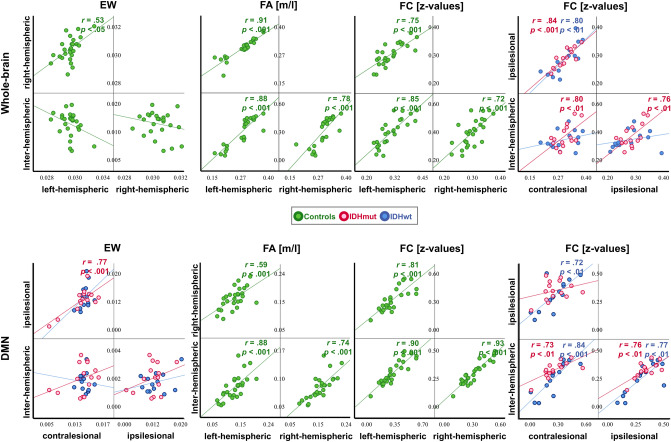
Intramodal structural and functional connectivity correlations. Significant intramodal correlations are visualized for edge weight (EW), fractional anisotropy (FA) and functional connectivity (FC) for healthy controls (green), and isodehydrogenase-mutated (IDHmut, pink), and IDH-wildtype gliomas (IDHwt, blue), and are marked with the correlation coefficient (r) and the significance (p). Significances for each analysis were computed two-sided with a significance level of *p* < .05 and corrected for multiple comparisons, corrected *p* value: *p* < .006.

Intermodal partial correlation analyses of SC and FC measures did not reach significance in any group. All significant correlations are contrasted for controls, IDHmut, and IDHwt in Fig. 5.

### Intra- and intermodal partial correlations of DMN SC and FC

In controls, partial correlation analyses revealed intramodal correlations within the structural (FA) and functional connectome, revealing left-and right-hemispheric connectivity measures to be positively correlated with each other and with interhemispheric connectivity (FA_LEFT_–FA_RIGHT_: *r*_partial_ = 0.59, *p* = 0.001; FA_LEFT_–FA_INTERHEM_: *r*_partial_ = 0.68, *p* = 0.000; FA_RIGHT_–FA_INTERHEM_: *r*_partial_ = 0.74, *p* = 0.000, FC_LEFT_–FC_RIGHT_: *r*_partial_ = 0.81, *p* = 0.000; FC_LEFT_–FC_INTERHEM_: *r*_partial_ = 0.90, *p* = 0.000; FC_RIGHT_–FC_INTERHEM_: *r*_partial_ = 0.93, *p* = 0.000).

In IDHmut patients, less intramodal SC correlations were found. In particular, only contra- and ipsi-tumoral EW measures correlated positively with each other (EW_CONTRA_–EW_IPSI_: *r*_partial_ = 0.77, *p* = 0.001). In addition, contra- and ipsi-tumoral FC measures were positively correlated with interhemispheric FC (FC_CONTRA_-FC_INTERHEM_: *r*_partial_ = 0.73, *p* = 0.003; FC_IPSI_–FC_INTERHEM_: *r*_partial_ = 0.76, *p* = 0.002).

In IDHwt patients however, no intramodal correlations of SC measures were found, while intramodal correlations within the functional connectome remained significant (FC_CONTRA_–FC_IPSI_: *r*_partial_ = 0.72, *p* = 0.005; FC_CONTRA_–FC_INTERHEM_: *r*_partial_ = 0.84, *p* = 0.000; FC_IPSI_–FC_INTERHEM_: *r*_partial_ = 0.77, *p* = 0.002).

Intermodal partial correlation analyses of SC and FC measures did not reach significance in any group.

## Discussion

In order to assess the systemic impact of glioma on cerebral SC and FC, we determined both the structural and functional connectome in glioma patients in comparison to healthy controls. While healthy controls showed strong intramodal correlations both for SC and FC, indicating strong within-connectomic coherence, patients showed in particular a loss in connectomic structural coherence, while functional connectomic coherence appeared to be better preserved. Moreover, signs of WM structural disintegrity were encountered only in the prognostically less favorable IDHwt patients, which showed a decrease in FA within the ipsilesional hemisphere, while FA was increased in IDHmut patients in the contralesional hemisphere.

We investigated connectomic profiles for whole-brain, but as well more specifically for a highly connected hub region, the DMN^1^, as glioma associated FC has previously been suggested to be altered with regard to hub and non-hub connectivity^5^, and, moreover, to differ depending on tumor grade. The DMN as intrinsic network is considered essential for information processing and integration of different neural network functions^1,42^, and is known to be inter- and intraindividually highly robust. As relevant parts of the DMN encompass midline structures, the overlap with tumor regions in our study sample was also limited. In glioma, FC alterations within the DMN have previously been related to cognitive functions^9,23^, and have been found to be influenced by lesion site and tumor grade^9,25,43^. In our study, functional connectomic coherence in IDHwt patients appeared to be preserved within the DMN, but reduced for whole-brain, with the reverse in IDHmut patients. This might relate to previously suggested^5^ glioma-associated changes in hub/hub, hub/non-hub and non-hub/non-hub connectivity, with alterations in connectomic profile having been suggested to be associated with tumor grade. Our findings of different weightings of functional connectomic coherence of hub regions in our patients could relate to this notion, in that patients with the more rapidly progressing IDHwt glioma type may have concentrated functional resources onto hub/hub connectivity, while in IDHmut tumor patients, hub/non-hub connectivity might have been more relevant. Alterations in WM microarchitecture and loss in structural connectomic coherence may have necessitated a functional reorganization of hub and non-hub communication in glioma patients.

Whole-brain FC aberrations in glioma patients have recently been described^44^ to relate to tumor grade and IDH-mutation status. Our findings agree with these observations, in that whole-brain functional connectomic coherence for whole-brain was reduced only in IDHwt patients.

Tumor associated changes in whole-brain FC were previously hypothesized to relate to systemic microstructural WM disintegration caused by occult tumor cell migration, or relate to neurovascular uncoupling due to tumor-associated changes in microvasculature with neurovascular uncoupling, resulting in BOLD response changes not necessarily related to neuronal function. Our findings support the notion that glioma associated alterations in FC are at least accompanied if not preceded by systemic alterations of WM microarchitecture, which may provide an anatomical basis for more widespread FC aberrations. SC measures in our study comprised FA and EW as a normalized measure of all streamlines connecting each parcellated region with one another, with EW having previously been suggested to be a more appropriate measure to quantify SC^45^. Under consideration of methodological constraints in fiber tracking due to peritumoral vasogenic edema, we applied an additional free water correction algorithm, which not only allowed to improve fiber tracking in perilesional regions with marked perifocal edema, but as well aided to differentiate diffusion anisotropy loss due to fiber degradation from signal loss due to free water^32^. Thereby, potential tumor-associated fiber reductions especially in the ipsilesional hemisphere may have been less prominent in the current analysis than could otherwise have been presumed. However, while EW gives an estimate of the macroscopic WM architecture, it does not necessarily reflect the functional integrity of the fibers tracked. By quantifying diffusion anisotropy, FA is regarded as additionally providing in vivo estimates of microstructural WM integrity, potentially reflecting variations in fiber diameter, degree of myelination, fiber density, or fiber integrity^46^. Albeit ultimately unspecific unless histologically proven, glioma associated FA alterations have previously been related to occult tumor cell infiltration and microstructural WM disintegration^13–15,17,19^.

In our study, interhemispheric EW for whole-brain was decreased in patients, which complies with the assumption of tumor associated interhemispheric SC disruptions, potentially mediating long-range FC alterations with associated functional (e.g. cognitive) impairment in tumor patients^4,8,24^. Although intrahemispheric EW did not differ across groups, signs of microstructural WM desintegrity as indicated by decreases in FA were found only in the prognostically less favorable IDHwt glioma patients. Under consideration of significant age differences within our cohort, it cannot completely be excluded that age-related microstructural WM alterations may have influenced the observed FA and EW differences between groups. Our findings however agree with previous studies showing systemic microstructural WM desintegrity in glioma patients^13,14,27^, and comply with the notion of a highly proliferative and more invasive nature of IDHwt gliomas^19,26^. Furthermore, the decrease of interhemispheric EW in both patient groups may not be expected, if sole age-related effects had defined the observed between-group differences^47^. Interestingly, IDHmut patients showed higher FA values in the contralesional hemisphere, not only compared to IDHwt gliomas, but as well as compared to healthy controls. The finding of increases in FA in the contra-lesional hemisphere in the prognostically more favorable IDHmut patient group might cautiously be considered in the context of neuroplasticity within the contralesional hemisphere, which is more likely to occur in those patients with slower tumor growth rates, in which functional abilities are generally better preserved^48,49^, even despite larger tumor volumes. Neuroplasticity with contralesional increases in homotopic grey matter volumes has in this regard been previously reported in lower grade gliomas^50^, which thus may as well be associated with corresponding WM alterations in the contralesional hemisphere, as indicated by our findings.

Intramodal correlations were analyzed in our study in order to assess within-connectomic coherence, which may relate to global integration of long-range information processing. Interestingly, only healthy subjects showed strong whole- brain intramodal correlations, both for SC and FC. In patients on the contrary, no intramodal correlations for any of the structural measures were found for whole-brain, while only for EW within the DMN in IDHmut patients. This supports the assumption of tumor-associated global structural alterations, suggesting a larger-scale connectomic disorganization in patients. With regard to intramodal FC correlations, functional connectomic coherence still seemed to be relatively better preserved than structural connectomic coherence in our patients, albeit less consistently as compared to healthy controls. This may relate to efforts of maintaining functions by long-range functional integration in the tumor lesioned brain. As opposed to acute brain damage, clinical manifestation of chronically progressive tumor lesions is likely to be preceded by continuous connectomic alterations, long before disease manifestation and before functional impairment surfaces. It thus has to be assumed that tumor characteristics like tumor site, -volume and -growth dynamics impact on the degree and spatio-temporal extent of connectomic alterations. Therefore inversely, structural and functional connectomic phenotyping of patients with focal tumor lesions may as well be informative on tumor biology and disease dynamics.

Nenning et al.^51^ recently showed in longitudinal rs-fMRI measurements of six patients with glioblastoma, that longitudinal whole-brain FC aberrations reflected even the disease course, with functional network deterioration preceding tumor reoccurrence by two months. Our study furthermore suggests that at the time of disease manifestation, structural connectomic aberrations might be more pronounced in glioma patients than functional connectomic alterations, which may relate to IDH-mutation status. Dissociation of structural and functional connectomic integrity might indicate neural network disintegration, and may aid to identify prognostically differing tumor patients. Structural and functional connectomic profiling in tumor patients therefore could provide information complementary to macroscopic properties of the primary lesion site, with connectomic alterations potentially accompanying or even preceding macroscopically apparent tumor growth dynamics.

With rs-fMRI and DTI tractography based measures of SC and FC having been previously shown to correlate in healthy subjects^52^, it may seem contradictory that no significant SC-FC correlations were found in the present study^52,53^. The fact, that SC-FC correlations did not reach significance in our study may in part be due to the small study sample, as well as due to methodological issues. For example, fiber tracking algorithms can be limited by fiber length and -curvature (particularly in transcallosal fiber trajectories) or fiber crossing, while functional MRI can be altered by neurovascular uncoupling in tumor patients, potentially resulting in a falsely negative BOLD-response. While SC-FC correlations have previously been found to strongly differ regionally^52^, it has to be noted that in our study analyses were based on intra- and interhemispheric mean connectivity values, whereby locoregional SC/FC variations may have been canceled out, leaving insufficient results in this regard. It has furthermore to be considered that only those regions with at least one streamline connection were included in our analyses, leaving out FC beyond these regions, as well as FC based on indirect SC^53^. Further studies with alternative methodological approaches and larger study samples are thus required to further elicit potential SC and FC associations -or dissociations- in these patients. We however chose the current approach in order to be able to compare different patient groups, while having to take into account varying lesion locations and -sizes. Thereby, we rather aimed at a characterization of general patterns of connectomic alterations with regard to tumor-near (ipsilesional), tumor-distant (contralesional) and interhemispheric connectivity in prognostically differing tumor patients.

A major limitation of our study is the small sample size, which prevented further subanalyses with regard to different lesion locations and further tumor specifications. Furthermore, tumor-induced alterations in microvasculature and related neurovascular uncoupling may have additionally altered the BOLD-response in tumor patients^54^, independent of WM characteristics. More homogenous patient collectives and larger sample sizes, as well as longitudinal observations are needed to gain a deeper understanding of tumor-induced connectomic alterations.

In summary, glioma patients showed a decrease mainly in structural connectomic coherence, with microstructural WM disintegrity additionally found in the prognostically less favorable IDHwt group. The dissociation of functional and structural connectomic coherence might be an indicator of tumor-related neural network disintegration, and may provide complementary information on tumor biology in glioma patients. Connectomic profiling may aid in phenotyping and monitoring prognostically differing tumor types.

## Supplementary Information

Supplementary Information 1.

Supplementary Information 2.

Supplementary Information 3.

## Data Availability

The datasets generated during and/or analyzed during the current study are not publicly available due to data privacy protection obligations but are available from the corresponding author on reasonable request.
