# Author Correction: Dissociation of structural and functional connectomic coherence in glioma patients

**DOI:** 10.1038/s41598-023-31975-0

**Published:** 2023-04-03

**Authors:** Kerstin Jütten, Leon Weninger, Verena Mainz, Siegfried Gauggel, Ferdinand Binkofski, Martin Wiesmann, Dorit Merhof, Hans Clusmann, Chuh‑Hyoun Na

**Affiliations:** 1 Department of Neurosurgery, RWTH Aachen University, Pauwelsstraße 30, 52074 Aachen, Germany; 2 Imaging and Computer Vision, RWTH Aachen University, Templergraben 55, 52074 Aachen, Germany; 3 Institute of Medical Psychology and Medical Sociology, RWTH Aachen University, Pauwelsstraße 19, 52074 Aachen, Germany; 4 Division of Clinical Cognitive Sciences, RWTH Aachen University, Pauwelsstraße 17, 52074 Aachen, Germany; 5 Department of Diagnostic and Interventional Neuroradiology, RWTH Aachen University, Pauwelsstraße 30, 52074 Aachen, Germany; 6 Center for Integrated Oncology Aachen Bonn Cologne Duesseldorf (CIO ABCD), Aachen, Germany

Correction to: *Scientific Reports* 10.1038/s41598-021-95932-5, published online 18 August 2021

The Supplementary Information 1 file published with this Article contained errors, where the information of two patients was omitted. The original Supplementary Information 1 file is provided below for the record.

The errors have now been corrected in the Supplementary Information 1 file that accompanies the original Article.

## Supplementary Information

Supplementary Information 1.

