# Supplementary material for: Dissociation of structural and functional connectomic coherence in glioma patients
Source: Sci Rep. 2021 Aug 18;11:16790. doi: 10.1038/s41598-021-95932-5 (PMC8373888; doi:10.1038/s41598-021-95932-5)
Supplement: Supplementary file 1 — Supplementary Information 1. [file 41598_2021_95932_MOESM1_ESM.docx]

**Supplement 1 Clinical description of included patients**

| **Patients** | **IDH-mutation** | **Diagnosis** | **Grade** | **Location** | **Side** | **Volume (in mm³)** | **Age (years)** | **Education (years)***** |
| --- | --- | --- | --- | --- | --- | --- | --- | --- |
| 1 | y | Oligodendroglioma* | II | Frontal | r | 2 | 36-40 | 13 |
| 2 | y | Oligodendroglioma* | II | Frontal | l | 24 | 26-30 | 13 |
| 3 | y | Astrocytoma | II | Insular | r | 30 | 30-35 | 13 |
| 4 | y | Astrocytoma | II | Insular | l | 32 | 30-35 | 15 |
| 5 | y | Astrocytoma | II | Frontal | l | 51 | 26-30 | 16 |
| 6 | y | Astrocytoma | II | Frontal | l | 54 | 26-30 | 13 |
| 7 | y | Astrocytoma | II | Parietal | l | 73 | 56-60 | 18 |
| 8 | y | Anaplastic astrocytoma | III | Frontal | l | 21 | 50-55 | 13 |
| 9 | y | Anaplastic astrocytoma | III | Parietal | r | 25 | 56-60 | 9 |
| 10 | y | Anaplastic oligodendroglioma* | III | Frontal | l | 39 | 50-55 | 15 |
| 11 | y | Anaplastic oligodendroglioma* | III | Frontal | r | 96 | 30-35 | 18 |
| 12 | y | Anaplastic astrocytoma | III | Temporal, parietal | l | 114 | 40-45 | 13 |
| 13 | y | Anaplastic astrocytoma | III | Parietal | l | 119 | 20-25 | 13 |
| 14 | y | Anaplastic astrocytoma | III | Frontal | r | 155 | 30-35 | 15 |
| 15 | y | Anaplastic oligodendroglioma* | III | Frontal | r | 175 | 30-35 | 13 |
| 16 | n | Anaplastic astrocytoma | III | Frontal | l | 49 | 40-45 | 13 |
| 17 | n | Anaplastic astrocytoma | III | Temporal, parietal | l | 51 | 70-75 | 18 |
| 18 | n | Glioblastoma multiforme | IV | Frontal | r | 19 | 66-70 | 12 |
| 19 | n | Glioblastoma multiforme | IV | Temporo-parietal | l | 2 | 56-60 | 10 |
| 20 | n | Glioblastoma multiforme** | IV | Frontal | l | 11 | 76-80 | 13 |
| 21 | n | Glioblastoma multiforme | IV | Temporo-parietal-occipital | l | 11 | 60-65 | 15 |
| 22 | n | Glioblastoma multiforme** | IV | Temporo-parietal | l | 13 | 50-55 | 13 |
| 23 | n | Glioblastoma multiforme | IV | Frontal, insular | l | 20 | 66-70 | 9 |
| 24 | n | Glioblastoma multiforme | IV | Temporal, parietal, occipital | l | 25 | 66-70 | 12 |
| 25 | n | Glioblastoma multiforme | IV | Occipital | l | 44 | 50-55 | 13 |
| 26 | n | Glioblastoma multiforme | IV | Parietal, occipital | r | 47 | 76-80 | 9 |
| 27 | n | Glioblastoma multiforme | IV | Parietal | l | 64 | 60-65 | 9 |
| 28 | n | Glioblastoma multiforme | IV | Temporal, parietal | l | 116 | 66-70 | 18 |
| 29 | n | Glioblastoma multiforme | IV | Frontal | r | 121 | 56-60 | 16 |

*Note*. IDH=isocitrate-dehydrogenase, y=yes, n=no, l=left, r=right. *Patients with codeletion of chromosome arms 1p and 19q. **Recurrent tumor with preceding tumor resection and adjuvant radiochemotherapy. ***Years of education were computed by the sum of years spent for school career and further training/study.
