# Supplementary material for: Dissociation of structural and functional connectomic coherence in glioma patients
Source: Sci Rep. 2021 Aug 18;11:16790. doi: 10.1038/s41598-021-95932-5 (PMC8373888; doi:10.1038/s41598-021-95932-5)
Supplement: Supplementary file 2 — Supplementary Information 2. [file 41598_2021_95932_MOESM2_ESM.docx]

**Supplement 2 Correlations between structural and functional connectivity parameters in controls and patient groups**

| Analysis | Variable | Significance | EW left | EW right | EW inter | FA left | FA right | FA inter | RS left | RS right | RS inter | subgroup |
| --- | --- | --- | --- | --- | --- | --- | --- | --- | --- | --- | --- | --- |
| Whole-brain | EW left | *r* | 1.00 | 0.53 | -0.33 | 0.24 | 0.30 | 0.00 | 0.29 | 0.27 | 0.28 | Controls |
|  |  | *p* |  | 0.01 | 0.10 | 0.23 | 0.14 | 0.98 | 0.15 | 0.18 | 0.17 |  |
|  | EW right | *r* | 0.53 | 1.00 | -0.19 | 0.23 | 0.30 | 0.09 | 0.23 | 0.28 | 0.37 |  |
|  |  | *p* | 0.01 |  | 0.35 | 0.25 | 0.14 | 0.65 | 0.27 | 0.16 | 0.07 |  |
|  | EW inter. | *r* | -0.33 | -0.19 | 1.00 | -0.09 | -0.03 | 0.25 | 0.12 | -0.27 | -0.06 |  |
|  |  | *p* | 0.10 | 0.35 |  | 0.67 | 0.89 | 0.22 | 0.57 | 0.18 | 0.76 |  |
|  | FA left | *r* | 0.24 | 0.23 | -0.09 | 1.00 | **0.91** | **0.88** | 0.01 | -0.07 | -0.02 |  |
|  |  | *p* | 0.23 | 0.25 | 0.67 |  | **0.00** | **0.00** | 0.95 | 0.74 | 0.93 |  |
|  | FA right | *r* | 0.30 | 0.30 | -0.03 | **0.91** | 1.00 | **0.78** | 0.08 | -0.04 | 0.05 |  |
|  |  | *p* | 0.14 | 0.14 | 0.89 | **0.00** |  | **0.00** | 0.70 | 0.84 | 0.80 |  |
|  | FA inter. | *r* | 0.00 | 0.09 | 0.25 | **0.88** | **0.78** | 1.00 | 0.02 | -0.13 | -0.08 |  |
|  |  | *p* | 0.98 | 0.65 | 0.22 | **0.00** | **0.00** |  | 0.94 | 0.53 | 0.71 |  |
|  | RS left | *r* | 0.29 | 0.23 | 0.12 | 0.01 | 0.08 | 0.02 | 1.00 | **0.75** | **0.84** |  |
|  |  | *p* | 0.15 | 0.27 | 0.57 | 0.95 | 0.70 | 0.94 |  | **0.00** | **0.00** |  |
|  | RS right | *r* | 0.27 | 0.28 | -0.27 | -0.07 | -0.04 | -0.13 | **0.75** | 1.00 | **0.72** |  |
|  |  | *p* | 0.18 | 0.16 | 0.18 | 0.74 | 0.84 | 0.53 | **0.00** |  | **0.00** |  |
|  | RS inter. | *r* | 0.28 | 0.37 | -0.06 | -0.02 | 0.05 | -0.08 | **0.84** | **0.72** | 1.00 |  |
|  |  | *p* | 0.17 | 0.07 | 0.76 | 0.93 | 0.80 | 0.71 | **0.00** | **0.00** |  |  |
| DMN | EW left | *r* | 1.00 | 0.51 | -0.29 | 0.53 | 0.33 | 0.42 | 0.12 | -0.06 | 0.01 |  |
|  |  | *p* |  | 0.01 | 0.15 | 0.01 | 0.10 | 0.03 | 0.55 | 0.79 | 0.95 |  |
|  | EW right | *r* | 0.51 | 1.00 | -0.09 | 0.45 | 0.51 | 0.35 | 0.00 | 0.03 | 0.08 |  |
|  |  | *p* | 0.01 |  | 0.65 | 0.02 | 0.01 | 0.08 | 0.99 | 0.88 | 0.70 |  |
|  | EW inter. | *r* | -0.29 | -0.09 | 1.00 | -0.05 | 0.07 | 0.00 | -0.42 | -0.38 | -0.38 |  |
|  |  | *p* | 0.15 | 0.65 |  | 0.81 | 0.74 | 0.99 | 0.03 | 0.06 | 0.06 |  |
|  | FA left | *r* | 0.53 | 0.45 | -0.05 | 1.00 | **0.59** | **0.68** | 0.28 | -0.01 | 0.17 |  |
|  |  | *p* | 0.01 | 0.02 | 0.81 |  | **0.00** | **0.00** | 0.17 | 0.97 | 0.41 |  |
|  | FA right | *r* | 0.33 | 0.51 | 0.07 | **0.59** | 1.00 | **0.74** | 0.04 | 0.02 | 0.05 |  |
|  |  | *p* | 0.10 | 0.01 | 0.74 | **0.00** |  | **0.00** | 0.84 | 0.93 | 0.80 |  |
|  | FA inter. | *r* | 0.42 | 0.35 | 0.00 | **0.68** | **0.74** | 1.00 | 0.15 | 0.04 | 0.12 |  |
|  |  | *p* | 0.03 | 0.08 | 0.99 | **0.00** | **0.00** |  | 0.48 | 0.86 | 0.56 |  |
|  | RS left | *r* | 0.12 | 0.00 | -0.42 | 0.28 | 0.04 | 0.15 | 1.00 | **0.81** | **0.90** |  |
|  |  | *p* | 0.55 | 0.99 | 0.03 | 0.17 | 0.84 | 0.48 |  | **0.00** | **0.00** |  |
|  | RS right | *r* | -0.06 | 0.03 | -0.38 | -0.01 | 0.02 | 0.04 | **0.81** | 1.00 | **0.93** |  |
|  |  | *p* | 0.79 | 0.88 | 0.06 | 0.97 | 0.93 | 0.86 | **0.00** |  | **0.00** |  |
|  | RS inter. | *r* | 0.01 | 0.08 | -0.38 | 0.17 | 0.05 | 0.12 | **0.90** | **0.93** | 1.00 |  |
|  |  | *p* | 0.95 | 0.70 | 0.06 | 0.41 | 0.80 | 0.56 | **0.00** | **0.00** |  |  |

*Note*. EW=edge weight, FA=fractional anisotropy, RS=resting state, r=correlation coefficient, p=significance, DMN=default-mode network.

| Analysis | Variable | Significance | EW contra | EW ipsi | EW inter | FA contra | FA ipsi | FA inter | RS contra | RS ipsi | RS inter | subgroup |
| --- | --- | --- | --- | --- | --- | --- | --- | --- | --- | --- | --- | --- |
| Whole-brain | EW left | *r* | 1.00 | 0.55 | 0.21 | 0.54 | 0.43 | 0.04 | 0.07 | 0.21 | -0.16 | IDHmut |
|  |  | *p* |  | 0.04 | 0.47 | 0.05 | 0.12 | 0.88 | 0.80 | 0.46 | 0.59 |  |
|  | EW right | *r* | 0.55 | 1.00 | -0.13 | 0.08 | 0.12 | 0.02 | 0.08 | 0.20 | 0.10 |  |
|  |  | *p* | 0.04 |  | 0.66 | 0.77 | 0.69 | 0.94 | 0.80 | 0.50 | 0.74 |  |
|  | EW inter. | *r* | 0.21 | -0.13 | 1.00 | -0.21 | -0.45 | 0.43 | 0.23 | 0.06 | -0.18 |  |
|  |  | *p* | 0.47 | 0.66 |  | 0.47 | 0.11 | 0.13 | 0.44 | 0.85 | 0.54 |  |
|  | FA left | *r* | 0.54 | 0.08 | -0.21 | 1.00 | 0.62 | -0.30 | -0.07 | -0.05 | -0.25 |  |
|  |  | *p* | 0.05 | 0.77 | 0.47 |  | 0.02 | 0.30 | 0.82 | 0.87 | 0.39 |  |
|  | FA right | *r* | 0.43 | 0.12 | -0.45 | 0.62 | 1.00 | -0.42 | -0.25 | -0.09 | -0.14 |  |
|  |  | *p* | 0.12 | 0.69 | 0.11 | 0.02 |  | 0.14 | 0.39 | 0.75 | 0.64 |  |
|  | FA inter. | *r* | 0.04 | 0.02 | 0.43 | -0.30 | -0.42 | 1.00 | 0.13 | 0.20 | 0.03 |  |
|  |  | *p* | 0.88 | 0.94 | 0.13 | 0.30 | 0.14 |  | 0.66 | 0.49 | 0.91 |  |
|  | RS left | *r* | 0.07 | 0.08 | 0.23 | -0.07 | -0.25 | 0.13 | 1.00 | **0.84** | **0.80** |  |
|  |  | *p* | 0.80 | 0.80 | 0.44 | 0.82 | 0.39 | 0.66 |  | **0.00** | **0.00** |  |
|  | RS right | *r* | 0.21 | 0.20 | 0.06 | -0.05 | -0.09 | 0.20 | **0.84** | 1.00 | **0.76** |  |
|  |  | *p* | 0.46 | 0.50 | 0.85 | 0.87 | 0.75 | 0.49 | **0.00** |  | **0.00** |  |
|  | RS inter. | *r* | -0.16 | 0.10 | -0.18 | -0.25 | -0.14 | 0.03 | **0.80** | **0.76** | 1.00 |  |
|  |  | *p* | 0.59 | 0.74 | 0.54 | 0.39 | 0.64 | 0.91 | **0.00** | **0.00** |  |  |
| DMN | EW left | *r* | 1.00 | **0.76** | 0.37 | 0.45 | 0.55 | 0.25 | 0.37 | -0.33 | 0.10 |  |
|  |  | *p* |  | **0.00** | 0.19 | 0.11 | 0.04 | 0.39 | 0.19 | 0.25 | 0.72 |  |
|  | EW right | *r* | **0.76** | 1.00 | 0.35 | 0.08 | 0.52 | 0.31 | 0.28 | -0.40 | 0.03 |  |
|  |  | *p* | **0.00** |  | 0.21 | 0.79 | 0.06 | 0.28 | 0.33 | 0.16 | 0.92 |  |
|  | EW inter. | *r* | 0.37 | 0.35 | 1.00 | 0.22 | -0.01 | 0.43 | 0.30 | -0.36 | 0.13 |  |
|  |  | *p* | 0.19 | 0.21 |  | 0.44 | 0.97 | 0.13 | 0.30 | 0.20 | 0.65 |  |
|  | FA left | *r* | 0.45 | 0.08 | 0.22 | 1.00 | 0.31 | 0.15 | -0.16 | -0.21 | -0.13 |  |
|  |  | *p* | 0.11 | 0.79 | 0.44 |  | 0.28 | 0.61 | 0.58 | 0.47 | 0.67 |  |
|  | FA right | *r* | 0.55 | 0.52 | -0.01 | 0.31 | 1.00 | 0.38 | 0.12 | -0.25 | -0.11 |  |
|  |  | *p* | 0.04 | 0.06 | 0.97 | 0.28 |  | 0.19 | 0.68 | 0.39 | 0.72 |  |
|  | FA inter. | *r* | 0.25 | 0.31 | 0.43 | 0.15 | 0.38 | 1.00 | -0.18 | -0.54 | -0.36 |  |
|  |  | *p* | 0.39 | 0.28 | 0.13 | 0.61 | 0.19 |  | 0.53 | 0.05 | 0.21 |  |
|  | RS left | *r* | 0.37 | 0.28 | 0.30 | -0.16 | 0.12 | -0.18 | 1.00 | 0.28 | **0.73** |  |
|  |  | *p* | 0.19 | 0.33 | 0.30 | 0.58 | 0.68 | 0.53 |  | 0.33 | **0.00** |  |
|  | RS right | *r* | -0.33 | -0.40 | -0.36 | -0.21 | -0.25 | -0.54 | 0.28 | 1.00 | **0.76** |  |
|  |  | *p* | 0.25 | 0.16 | 0.20 | 0.47 | 0.39 | 0.05 | 0.33 |  | **0.00** |  |
|  | RS inter. | *r* | 0.10 | 0.03 | 0.13 | -0.13 | -0.11 | -0.36 | **0.73** | **0.76** | 1.00 |  |
|  |  | *p* | 0.72 | 0.92 | 0.65 | 0.67 | 0.72 | 0.21 | **0.00** | **0.00** |  |  |

*Note*. EW=edge weight, FA=fractional anisotropy, RS=resting state, r=correlation coefficient, p=significance, DMN=default-mode network, IDH=isocitrate-dehydrogenase, IDHmut=IDH mutated glioma.

| Analysis | Variable | Significance | EW contra | EW ipsi | EW inter | FA contra | FA ipsi | FA inter. | RS contra | RS ipsi | RS inter | subgroup |
| --- | --- | --- | --- | --- | --- | --- | --- | --- | --- | --- | --- | --- |
| Whole-brain | EW left | *r* | 1.00 | 0.68 | -0.07 | -0.25 | 0.17 | -0.11 | -0.27 | -0.43 | -0.11 | IDHwt |
|  |  | *p* |  | 0.01 | 0.83 | 0.42 | 0.58 | 0.71 | 0.38 | 0.14 | 0.73 |  |
|  | EW right | *r* | 0.68 | 1.00 | -0.36 | -0.24 | 0.17 | -0.14 | -0.20 | -0.11 | 0.08 |  |
|  |  | *p* | 0.01 |  | 0.22 | 0.43 | 0.57 | 0.64 | 0.52 | 0.72 | 0.78 |  |
|  | EW inter. | *r* | -0.07 | -0.36 | 1.00 | 0.23 | 0.18 | 0.68 | 0.46 | 0.41 | 0.28 |  |
|  |  | *p* | 0.83 | 0.22 |  | 0.45 | 0.55 | 0.01 | 0.12 | 0.17 | 0.35 |  |
|  | FA left | *r* | -0.25 | -0.24 | 0.23 | 1.00 | 0.35 | 0.29 | 0.30 | 0.39 | -0.05 |  |
|  |  | *p* | 0.42 | 0.43 | 0.45 |  | 0.24 | 0.33 | 0.32 | 0.19 | 0.86 |  |
|  | FA right | *r* | 0.17 | 0.17 | 0.18 | 0.35 | 1.00 | 0.53 | -0.38 | -0.23 | -0.06 |  |
|  |  | *p* | 0.58 | 0.57 | 0.55 | 0.24 |  | 0.06 | 0.21 | 0.44 | 0.85 |  |
|  | FA inter. | *r* | -0.11 | -0.14 | 0.68 | 0.29 | 0.53 | 1.00 | 0.27 | 0.45 | 0.07 |  |
|  |  | *p* | 0.71 | 0.64 | 0.01 | 0.33 | 0.06 |  | 0.37 | 0.12 | 0.83 |  |
|  | RS left | *r* | -0.27 | -0.20 | 0.46 | 0.30 | -0.38 | 0.27 | 1.00 | **0.80** | 0.30 |  |
|  |  | *p* | 0.38 | 0.52 | 0.12 | 0.32 | 0.21 | 0.37 |  | **0.00** | 0.31 |  |
|  | RS right | *r* | -0.43 | -0.11 | 0.41 | 0.39 | -0.23 | 0.45 | **0.80** | 1.00 | 0.26 |  |
|  |  | *p* | 0.14 | 0.72 | 0.17 | 0.19 | 0.44 | 0.12 | **0.00** |  | 0.39 |  |
|  | RS inter. | *r* | -0.11 | 0.08 | 0.28 | -0.05 | -0.06 | 0.07 | 0.30 | 0.26 | 1.00 |  |
|  |  | *p* | 0.73 | 0.78 | 0.35 | 0.86 | 0.85 | 0.83 | 0.31 | 0.39 |  |  |
| DMN | EW left | *r* | 1.00 | 0.48 | 0.10 | -0.16 | 0.05 | 0.60 | 0.51 | 0.36 | 0.56 |  |
|  |  | *p* |  | 0.10 | 0.74 | 0.59 | 0.87 | 0.03 | 0.07 | 0.23 | 0.05 |  |
|  | EW right | *r* | 0.48 | 1.00 | 0.30 | -0.50 | 0.46 | 0.36 | 0.38 | 0.41 | 0.59 |  |
|  |  | *p* | 0.10 |  | 0.33 | 0.08 | 0.11 | 0.23 | 0.20 | 0.16 | 0.03 |  |
|  | EW inter. | *r* | 0.10 | 0.30 | 1.00 | 0.14 | 0.09 | 0.23 | -0.52 | -0.45 | -0.36 |  |
|  |  | *p* | 0.74 | 0.33 |  | 0.65 | 0.78 | 0.45 | 0.07 | 0.12 | 0.23 |  |
|  | FA left | *r* | -0.16 | -0.50 | 0.14 | 1.00 | 0.28 | 0.32 | -0.61 | -0.38 | -0.64 |  |
|  |  | *p* | 0.59 | 0.08 | 0.65 |  | 0.36 | 0.29 | 0.03 | 0.20 | 0.02 |  |
|  | FA right | *r* | 0.05 | 0.46 | 0.09 | 0.28 | 1.00 | 0.67 | 0.03 | 0.35 | 0.15 |  |
|  |  | *p* | 0.87 | 0.11 | 0.78 | 0.36 |  | 0.01 | 0.91 | 0.23 | 0.63 |  |
|  | FA inter. | *r* | 0.60 | 0.36 | 0.23 | 0.32 | 0.67 | 1.00 | 0.11 | 0.24 | 0.13 |  |
|  |  | *p* | 0.03 | 0.23 | 0.45 | 0.29 | 0.01 |  | 0.71 | 0.43 | 0.67 |  |
|  | RS left | *r* | 0.51 | 0.38 | -0.52 | -0.61 | 0.03 | 0.11 | 1.00 | **0.72** | **0.84** |  |
|  |  | *p* | 0.07 | 0.20 | 0.07 | 0.03 | 0.91 | 0.71 |  | **0.01** | **0.00** |  |
|  | RS right | *r* | 0.36 | 0.41 | -0.45 | -0.38 | 0.35 | 0.24 | **0.72** | 1.00 | **0.77** |  |
|  |  | *p* | 0.23 | 0.16 | 0.12 | 0.20 | 0.23 | 0.43 | **0.01** |  | **0.00** |  |
|  | RS inter. | *r* | 0.56 | 0.59 | -0.36 | -0.64 | 0.15 | 0.13 | **0.84** | **0.77** | 1.00 |  |
|  |  | *p* | 0.05 | 0.03 | 0.23 | 0.02 | 0.63 | 0.67 | **0.00** | **0.00** |  |  |

*Note*. EW=edge weight, FA=fractional anisotropy, RS=resting state, r=correlation coefficient, p=significance, DMN=default-mode network, IDH=isocitrate-dehydrogenase, IDHwt=IDH wildtype glioma.
