# Supplementary material for: Dissociation of structural and functional connectomic coherence in glioma patients
Source: Sci Rep. 2021 Aug 18;11:16790. doi: 10.1038/s41598-021-95932-5 (PMC8373888; doi:10.1038/s41598-021-95932-5)
Supplement: Supplementary file 3 — Supplementary Information 3. [file 41598_2021_95932_MOESM3_ESM.docx]

| **Lobe** | **Gyrus** | **Left and Right Hemisphere** | **Label ID.L** | **Label ID.R** | **Anatomical and modified Cyto-architectonic descriptions** | **lh.MNI(X,Y,Z)** | **rh.MNI(X,Y,Z)** |
| --- | --- | --- | --- | --- | --- | --- | --- |
| **Frontal Lobe** | SFG, Superior Frontal Gyrus | SFG_L(R)_7_1 | 1 | 2 | *A8m, medial area 8* | -5 ,15, 54 | 7, 16, 54 |
|  |  | SFG_L(R)_7_2 | 3 | 4 | *A8dl, dorsolateral area 8* | -18, 24, 53 | 22, 26, 51 |
|  |  | SFG_L(R)_7_3 | 5 | 6 | *A9l, lateral area 9* | -11, 49, 40 | 13, 48, 40 |
|  |  | SFG_L(R)_7_4 | 7 | 8 | *A6dl, dorsolateral area 6* | -18, -1, 65 | 20, 4, 64 |
|  |  | SFG_L(R)_7_5 | 9 | 10 | *A6m, medial area 6* | -6, -5, 58 | 7, -4, 60 |
|  |  | SFG_L(R)_7_6 | 11 | 12 | *A9m,medial area 9* | -5, 36, 38 | 6, 38, 35 |
|  |  | SFG_L(R)_7_7 | 13 | 14 | *A10m, medial area 10* | -8, 56, 15 | 8, 58, 13 |
|  | MFG, Middle Frontal Gyrus | MFG_L(R)_7_1 | 15 | 16 | *A9/46d, dorsal area 9/46* | -27, 43, 31 | 30, 37, 36 |
|  |  | MFG_L(R)_7_2 | 17 | 18 | *IFJ, inferior frontal junction* | -42, 13, 36 | 42, 11, 39 |
|  |  | MFG_L(R)_7_3 | 19 | 20 | *A46, area 46* | -28, 56, 12 | 28, 55, 17 |
|  |  | MFG_L(R)_7_4 | 21 | 22 | *A9/46v, ventral area 9/46* | -41, 41, 16 | 42, 44, 14 |
|  |  | MFG_L(R)_7_5 | 23 | 24 | *A8vl, ventrolateral area 8* | -33, 23, 45 | 42, 27, 39 |
|  |  | MFG_L(R)_7_6 | 25 | 26 | *A6vl, ventrolateral area 6* | -32, 4, 55 | 34, 8, 54 |
|  |  | MFG_L(R)_7_7 | 27 | 28 | *A10l, lateral area10* | -26, 60, -6 | 25, 61, -4 |
|  | IFG, Inferior Frontal Gyrus | IFG_L(R)_6_1 | 29 | 30 | *A44d,dorsal area 44* | -46, 13, 24 | 45, 16, 25 |
|  |  | IFG_L(R)_6_2 | 31 | 32 | *IFS, inferior frontal sulcus* | -47, 32, 14 | 48, 35, 13 |
|  |  | IFG_L(R)_6_3 | 33 | 34 | *A45c, caudal area 45* | -53, 23, 11 | 54, 24, 12 |
|  |  | IFG_L(R)_6_4 | 35 | 36 | *A45r, rostral area 45* | -49, 36, -3 | 51, 36, -1 |
|  |  | IFG_L(R)_6_5 | 37 | 38 | *A44op, opercular area 44* | -39, 23, 4 | 42, 22, 3 |
|  |  | IFG_L(R)_6_6 | 39 | 40 | *A44v, ventral area 44* | -52, 13, 6 | 54, 14, 11 |
|  | OrG, Orbital Gyrus | OrG_L(R)_6_1 | 41 | 42 | *A14m, medial area 14* | -7, 54, -7 | 6, 47, -7 |
|  |  | OrG_L(R)_6_2 | 43 | 44 | *A12/47o, orbital area 12/47* | -36, 33, -16 | 40, 39, -14 |
|  |  | OrG_L(R)_6_3 | 45 | 46 | *A11l, lateral area 11* | -23, 38, -18 | 23, 36, -18 |
|  |  | OrG_L(R)_6_4 | 47 | 48 | *A11m, medial area 11* | -6, 52, -19 | 6, 57, -16 |
|  |  | OrG_L(R)_6_5 | 49 | 50 | *A13, area 13* | -10, 18, -19 | 9, 20, -19 |
|  |  | OrG_L(R)_6_6 | 51 | 52 | *A12/47l, lateral area 12/47* | -41, 32, -9 | 42, 31, -9 |
|  | PrG, Precentral Gyrus | PrG_L(R)_6_1 | 53 | 54 | *A4hf, area 4(head and face region)* | -49, -8, 39 | 55, -2, 33 |
|  |  | PrG_L(R)_6_2 | 55 | 56 | *A6cdl, caudal dorsolateral area 6* | -32, -9, 58 | 33, -7, 57 |
|  |  | PrG_L(R)_6_3 | 57 | 58 | *A4ul, area 4(upper limb region)* | -26, -25, 63 | 34, -19, 59 |
|  |  | PrG_L(R)_6_4 | 59 | 60 | *A4t, area 4(trunk region)* | -13, -20, 73 | 15, -22, 71 |
|  |  | PrG_L(R)_6_5 | 61 | 62 | *A4tl, area 4(tongue and larynx region)* | -52, 0, 8 | 54, 4, 9 |
|  |  | PrG_L(R)_6_6 | 63 | 64 | *A6cvl, caudal ventrolateral area 6* | -49, 5, 30 | 51, 7, 30 |
|  | PCL, Paracentral Lobule | PCL_L(R)_2_1 | 65 | 66 | *A1/2/3ll, area1/2/3 (lower limb region)* | -8, -38, 58 | 10, -34, 54 |
|  |  | PCL_L(R)_2_2 | 67 | 68 | *A4ll, area 4, (lower limb region)* | -4, -23, 61 | 5, -21, 61 |
| **Temporal Lobe** | STG, Superior Temporal Gyrus | STG_L(R)_6_1 | 69 | 70 | *A38m, medial area 38* | -32, 14, -34 | 31, 15, -34 |
|  |  | STG_L(R)_6_2 | 71 | 72 | *A41/42, area 41/42* | -54, -32, 12 | 54, -24, 11 |
|  |  | STG_L(R)_6_3 | 73 | 74 | *TE1.0 and TE1.2* | -50, -11, 1 | 51, -4, -1 |
|  |  | STG_L(R)_6_4 | 75 | 76 | *A22c, caudal area 22* | -62, -33, 7 | 66, -20, 6 |
|  |  | STG_L(R)_6_5 | 77 | 78 | *A38l, lateral area 38* | -45, 11, -20 | 47, 12, -20 |
|  |  | STG_L(R)_6_6 | 79 | 80 | *A22r, rostral area 22* | -55, -3, -10 | 56, -12, -5 |
|  | MTG, Middle Temporal Gyrus | MTG_L(R)_4_1 | 81 | 82 | *A21c, caudal area 21* | -65, -30, -12 | 65, -29, -13 |
|  |  | MTG_L(R)_4_2 | 83 | 84 | *A21r, rostral area 21* | -53, 2, -30 | 51, 6, -32 |
|  |  | MTG_L(R)_4_3 | 85 | 86 | *A37dl, dorsolateral area37* | -59, -58, 4 | 60, -53, 3 |
|  |  | MTG_L(R)_4_4 | 87 | 88 | *aSTS, anterior superior temporal sulcus* | -58, -20, -9 | 58, -16, -10 |
|  | ITG, Inferior Temporal Gyrus | ITG_L(R)_7_1 | 89 | 90 | *A20iv, intermediate ventral area 20* | -45, -26, -27 | 46, -14, -33 |
|  |  | ITG_L(R)_7_2 | 91 | 92 | *A37elv, extreme lateroventral area37* | -51, -57, -15 | 53, -52, -18 |
|  |  | ITG_L(R)_7_3 | 93 | 94 | *A20r, rostral area 20* | -43, -2, -41 | 40, 0, -43 |
|  |  | ITG_L(R)_7_4 | 95 | 96 | *A20il, intermediate lateral area 20* | -56, -16, -28 | 55, -11, -32 |
|  |  | ITG_L(R)_7_5 | 97 | 98 | *A37vl, ventrolateral area 37* | -55, -60, -6 | 54, -57, -8 |
|  |  | ITG_L(R)_7_6 | 99 | 100 | *A20cl, caudolateral of area 20* | -59, -42, -16 | 61, -40, -17 |
|  |  | ITG_L(R)_7_7 | 101 | 102 | *A20cv, caudoventral of area 20* | -55, -31, -27 | 54, -31, -26 |
|  | FuG, Fusiform Gyrus | FuG_L(R)_3_1 | 103 | 104 | *A20rv, rostroventral area 20* | -33, -16, -32 | 33, -15, -34 |
|  |  | FuG_L(R)_3_2 | 105 | 106 | *A37mv, medioventral area37* | -31, -64, -14 | 31, -62, -14 |
|  |  | FuG_L(R)_3_3 | 107 | 108 | *A37lv, lateroventral area37* | -42, -51, -17 | 43, -49, -19 |
|  | PhG, Parahippocampal Gyrus | PhG_L(R)_6_1 | 109 | 110 | *A35/36r, rostral area 35/36* | -27, -7, -34 | 28, -8, -33 |
|  |  | PhG_L(R)_6_2 | 111 | 112 | *A35/36c, caudal area 35/36* | -25, -25, -26 | 26, -23, -27 |
|  |  | PhG_L(R)_6_3 | 113 | 114 | *TL, area TL (lateral PPHC, posterior parahippocampal gyrus)* | -28, -32, -18 | 30, -30, -18 |
|  |  | PhG_L(R)_6_4 | 115 | 116 | *A28/34, area 28/34 (EC, entorhinal cortex)* | -19, -12, -30 | 19, -10, -30 |
|  |  | PhG_L(R)_6_5 | 117 | 118 | *TI, area TI(temporal agranular insular cortex)* | -23, 2, -32 | 22, 1, -36 |
|  |  | PhG_L(R)_6_6 | 119 | 120 | *TH, area TH (medial PPHC)* | -17, -39, -10 | 19, -36, -11 |
|  | pSTS, posterior Superior Temporal Sulcus | pSTS_L(R)_2_1 | 121 | 122 | *rpSTS, rostroposterior superior temporal sulcus* | -54, -40, 4 | 53, -37, 3 |
|  |  | pSTS_L(R)_2_2 | 123 | 124 | *cpSTS, caudoposterior superior temporal sulcus* | -52, -50, 11 | 57, -40, 12 |
| **Parietal Lobe** | SPL, Superior Parietal Lobule | SPL_L(R)_5_1 | 125 | 126 | *A7r, rostral area 7* | -16, -60, 63 | 19, -57, 65 |
|  |  | SPL_L(R)_5_2 | 127 | 128 | *A7c, caudal area 7* | -15, -71, 52 | 19, -69, 54 |
|  |  | SPL_L(R)_5_3 | 129 | 130 | *A5l, lateral area 5* | -33, -47, 50 | 35, -42, 54 |
|  |  | SPL_L(R)_5_4 | 131 | 132 | *A7pc, postcentral area 7* | -22, -47, 65 | 23, -43, 67 |
|  |  | SPL_L(R)_5_5 | 133 | 134 | *A7ip, intraparietal area 7(hIP3)* | -27, -59, 54 | 31, -54, 53 |
|  | IPL, Inferior Parietal Lobule | IPL_L(R)_6_1 | 135 | 136 | *A39c, caudal area 39(PGp)* | -34, -80, 29 | 45, -71, 20 |
|  |  | IPL_L(R)_6_2 | 137 | 138 | *A39rd, rostrodorsal area 39(Hip3)* | -38, -61, 46 | 39, -65, 44 |
|  |  | IPL_L(R)_6_3 | 139 | 140 | *A40rd, rostrodorsal area 40(PFt)* | -51, -33, 42 | 47, -35, 45 |
|  |  | IPL_L(R)_6_4 | 141 | 142 | *A40c, caudal area 40(PFm)* | -56, -49, 38 | 57, -44, 38 |
|  |  | IPL_L(R)_6_5 | 143 | 144 | *A39rv, rostroventral area 39(PGa)* | -47, -65, 26 | 53, -54, 25 |
|  |  | IPL_L(R)_6_6 | 145 | 146 | *A40rv, rostroventral area 40(PFop)* | -53, -31, 23 | 55, -26, 26 |
|  | Pcun, Precuneus | PCun_L(R)_4_1 | 147 | 148 | *A7m, medial area 7(PEp)* | -5, -63, 51 | 6, -65, 51 |
|  |  | PCun_L(R)_4_2 | 149 | 150 | *A5m, medial area 5(PEm)* | -8, -47, 57 | 7, -47, 58 |
|  |  | PCun_L(R)_4_3 | 151 | 152 | *dmPOS, dorsomedial parietooccipital sulcus(PEr)* | -12, -67, 25 | 16, -64, 25 |
|  |  | PCun_L(R)_4_4 | 153 | 154 | *A31, area 31 (Lc1)* | -6, -55, 34 | 6, -54, 35 |
|  | PoG, Postcentral Gyrus | PoG_L(R)_4_1 | 155 | 156 | *A1/2/3ulhf, area 1/2/3(upper limb, head and face region)* | -50, -16, 43 | 50, -14, 44 |
|  |  | PoG_L(R)_4_2 | 157 | 158 | *A1/2/3tonIa, area 1/2/3(tongue and larynx region)* | -56, -14, 16 | 56, -10, 15 |
|  |  | PoG_L(R)_4_3 | 159 | 160 | *A2, area 2* | -46, -30, 50 | 48, -24, 48 |
|  |  | PoG_L(R)_4_4 | 161 | 162 | *A1/2/3tru, area1/2/3(trunk region)* | -21, -35, 68 | 20, -33, 69 |
| **Insular Lobe** | INS, Insular Gyrus | INS_L(R)_6_1 | 163 | 164 | *G, hypergranular insula* | -36, -20, 10 | 37, -18, 8 |
|  |  | INS_L(R)_6_2 | 165 | 166 | *vIa, ventral agranular insula* | -32, 14, -13 | 33, 14, -13 |
|  |  | INS_L(R)_6_3 | 167 | 168 | *dIa, dorsal agranular insula* | -34, 18, 1 | 36, 18, 1 |
|  |  | INS_L(R)_6_4 | 169 | 170 | *vId/vIg, ventral dysgranular and granular insula* | -38, -4, -9 | 39, -2, -9 |
|  |  | INS_L(R)_6_5 | 171 | 172 | *dIg, dorsal granular insula* | -38, -8, 8 | 39, -7, 8 |
|  |  | INS_L(R)_6_6 | 173 | 174 | *dId, dorsal dysgranular insula* | -38, 5, 5 | 38, 5, 5 |
| **Limbic Lobe** | CG, Cingulate Gyrus | CG_L(R)_7_1 | 175 | 176 | *A23d, dorsal area 23* | -4, -39, 31 | 4, -37, 32 |
|  |  | CG_L(R)_7_2 | 177 | 178 | *A24rv, rostroventral area 24* | -3, 8, 25 | 5, 22, 12 |
|  |  | CG_L(R)_7_3 | 179 | 180 | *A32p, pregenual area 32* | -6, 34, 21 | 5, 28, 27 |
|  |  | CG_L(R)_7_4 | 181 | 182 | *A23v, ventral area 23* | -8, -47, 10 | 9, -44, 11 |
|  |  | CG_L(R)_7_5 | 183 | 184 | *A24cd, caudodorsal area 24* | -5, 7, 37 | 4, 6, 38 |
|  |  | CG_L(R)_7_6 | 185 | 186 | *A23c, caudal area 23* | -7, -23, 41 | 6, -20, 40 |
|  |  | CG_L(R)_7_7 | 187 | 188 | *A32sg, subgenual area 32* | -4, 39, -2 | 5, 41, 6 |
| **Occipital Lobe** | MVOcC*,* MedioVentral Occipital Cortex | MVOcC _L(R)_5_1 | 189 | 190 | *cLinG, caudal lingual gyrus* | -11, -82, -11 | 10, -85, -9 |
|  |  | MVOcC _L(R)_5_2 | 191 | 192 | *rCunG, rostral cuneus gyrus* | -5, -81, 10 | 7, -76, 11 |
|  |  | MVOcC _L(R)_5_3 | 193 | 194 | *cCunG, caudal cuneus gyrus* | -6, -94, 1 | 8, -90, 12 |
|  |  | MVOcC _L(R)_5_4 | 195 | 196 | *rLinG, rostral lingual gyrus* | -17, -60, -6 | 18, -60, -7 |
|  |  | MVOcC _L(R)_5_5 | 197 | 198 | *vmPOS,ventromedial parietooccipital sulcus* | -13, -68, 12 | 15, -63, 12 |
|  | LOcC, lateral Occipital Cortex | LOcC_L(R)_4_1 | 199 | 200 | *mOccG, middle occipital gyrus* | -31, -89, 11 | 34, -86, 11 |
|  |  | LOcC _L(R)_4_2 | 201 | 202 | *V5/MT+, area V5/MT+* | -46, -74, 3 | 48, -70, -1 |
|  |  | LOcC _L(R)_4_3 | 203 | 204 | *OPC, occipital polar cortex* | -18, -99, 2 | 22, -97, 4 |
|  |  | LOcC_L(R)_4_4 | 205 | 206 | *iOccG, inferior occipital gyrus* | -30, -88, -12 | 32, -85, -12 |
|  |  | LOcC _L(R)_2_1 | 207 | 208 | *msOccG, medial superior occipital gyrus* | -11, -88, 31 | 16, -85, 34 |
|  |  | LOcC _L(R)_2_2 | 209 | 210 | *lsOccG, lateral superior occipital gyrus* | -22, -77, 36 | 29, -75, 36 |
| **Subcortical Nuclei** | Amyg, Amygdala | Amyg_L(R)_2_1 | 211 | 212 | *mAmyg, medial amygdala* | -19, -2, -20 | 19, -2, -19 |
|  |  | Amyg_L(R)_2_2 | 213 | 214 | *lAmyg, lateral amygdala* | -27, -4, -20 | 28, -3, -20 |
|  | Hipp, Hippocampus | Hipp_L(R)_2_1 | 215 | 216 | *rHipp, rostral hippocampus* | -22, -14, -19 | 22, -12, -20 |
|  |  | Hipp_L(R)_2_2 | 217 | 218 | *cHipp, caudal hippocampus* | -28, -30, -10 | 29, -27, -10 |
|  | BG, Basal Ganglia | BG_L(R)_6_1 | 219 | 220 | *vCa, ventral caudate* | -12, 14, 0 | 15, 14, -2 |
|  |  | BG_L(R)_6_2 | 221 | 222 | *GP, globus pallidus* | -22, -2, 4 | 22, -2, 3 |
|  |  | BG_L(R)_6_3 | 223 | 224 | *NAC, nucleus accumbens* | -17, 3, -9 | 15, 8, -9 |
|  |  | BG_L(R)_6_4 | 225 | 226 | *vmPu, ventromedial putamen* | -23, 7, -4 | 22, 8, -1 |
|  |  | BG_L(R)_6_5 | 227 | 228 | *dCa, dorsal caudate* | -14, 2, 16 | 14, 5, 14 |
|  |  | BG_L(R)_6_6 | 229 | 230 | *dlPu, dorsolateral putamen* | -28, -5, 2 | 29, -3, 1 |
|  | Tha, Thalamus | Tha_L(R)_8_1 | 231 | 232 | *mPFtha, medial pre-frontal thalamus* | -7, -12, 5 | 7, -11, 6 |
|  |  | Tha_L(R)_8_2 | 233 | 234 | *mPMtha, pre-motor thalamus* | -18, -13, 3 | 12, -14, 1 |
|  |  | Tha_L(R)_8_3 | 235 | 236 | *Stha, sensory thalamus* | -18, -23, 4 | 18, -22, 3 |
|  |  | Tha_L(R)_8_4 | 237 | 238 | *rTtha, rostral temporal thalamus* | -7, -14, 7 | 3, -13, 5 |
|  |  | Tha_L(R)_8_5 | 239 | 240 | *PPtha, posterior parietal thalamus* | -16, -24, 6 | 15, -25, 6 |
|  |  | Tha_L(R)_8_6 | 241 | 242 | *Otha, occipital thalamus* | -15, -28, 4 | 13, -27, 8 |
|  |  | Tha_L(R)_8_7 | 243 | 244 | *cTtha, caudal temporal thalamus* | -12, -22, 13 | 10, -14, 14 |
|  |  | Tha_L(R)_8_8 | 245 | 246 | *lPFtha, lateral pre-frontal thalamus* | -11, -14, 2 | 13, -16, 7 |

*Note*. This table corresponds to file “[BNA_subregions.xlsx](https://pan.cstcloud.cn/s/6eRCJ0zDTFk)“ downloaded from the Brainnetome Atlas homepage (<https://atlas.brainnetome.org/download.html>.)
