# Supplementary material for: Author Correction: Dissociation of structural and functional connectomic coherence in glioma patients
Source: Sci Rep. 2023 Apr 3;13:5455. doi: 10.1038/s41598-023-31975-0 (PMC10070241; doi:10.1038/s41598-023-31975-0)
Supplement: Supplementary file 1 — Supplementary Information 1. [file 41598_2023_31975_MOESM1_ESM.docx]

**Supplement 1 Clinical description of included patients**

| Patients | IDH-mutation | Diagnosis | Grade | Location | Side | Volume (in cm³) | Age (years) | Education (years) |
| --- | --- | --- | --- | --- | --- | --- | --- | --- |
| 1 | y | Astrocytoma | II | Temporal | r | 30 | 30-35 | 13 |
| 2 | y | Astrocytoma | II | Frontal | l | 51 | 20-25 | 16 |
| 3 | y | Astrocytoma | II | Parietal | l | 64 | 55-60 | 18 |
| 4 | y | Astrocytoma | II | Frontal | l | 158 | 26-30 | 13 |
| 5 | y | Oligodendro-glioma | II | Frontal | r | 2 | 36-40 | 13 |
| 6 | y | Oligodendro-glioma | II | Frontal | l | 22 | 26-30 | 13 |
| 7 | y | Anaplastic astrocytoma^a^ | III | Frontal | r | 30 | 36-40 | 16 |
| 8 | y | Anaplastic astrocytoma | III | Parietal | l | 114 | 40-45 | 13 |
| 9 | y |  | III | Frontal | l | 49 | 40-45 | 13 |
| 10 | y | Anaplastic astrocytoma | III | Frontal | l | 21 | 50-55 | 13 |
| 11 | y | Anaplastic astrocytoma | III | Parietal | l | 119 | 20-25 | 13 |
| 12 | y | Anaplastic astrocytoma | III | Frontal | r | 155 | 30-35 | 15 |
| 13 | y | Anaplastic astrocytoma | III | Frontal, insular | r | 175 | 30-35 | 13 |
| 14 | y | Anaplastic oligodendro-glioma | III | Frontal | l | 39 | 50-55 | 15 |
| 15 | y | Anaplastic oligodendro-glioma | III | Frontal | r | 96 | 30-35 | 18 |
| 17 | n | Anaplastic astrocytoma | III | Temporo-mesial | l | 51 | 70-75 | 18 |
| 18 | n | Anaplastic astrocytoma | III | Parietal | r | 25 | 56-60 | 9 |
| 19 | n | Anaplastic astrocytoma | III | Frontal | l | 11 | 60-65 | 15 |
| 16 | n | Glioblastoma multiforme | IV | Occipital | l | 23 | 66-70 | 12 |
| 20 | n | Glioblastoma multiforme | IV | Fronto-parietal | l | 11 | 76-80 | 13 |
| 21 | n | Glioblastoma multiforme | IV | Temporal, insular | l | 111 | 56-60 | 10 |
| 22 | n | Glioblastoma multiforme | IV | Fronto-temporal, insular | l | 145 | 66-70 | 9 |
| 23 | n | Glioblastoma multiforme | IV | Occipital | l | 44 | 50-55 | 13 |
| 24 | n | Glioblastoma multiforme^a^ | IV | Temporo-parietal | l | 13 | 50-55 | 13 |
| 25 | n | Glioblastoma multiforme | IV | Frontal | r | 19 | 66-70 | 12 |
| 26 | n | Glioblastoma multiforme | IV | Frontal | r | 121 | 56-60 | 16 |
| 27 | n | Glioblastoma multiforme | IV | Occipital | r | 50 | 76-80 | 9 |

*Note*. This table corresponds to a table previously published by the authors{Jutten, 2020 #224}{Jütten, 2020 #209} [25]. IDH=isocitrate-dehydrogenase, y=yes, n=no, l=left, r=right, AE=anti-epileptics, m=male, f=female. ^a^Recurrent tumor with preceding tumor resection and adjuvant radiochemotherapy. *Years of education were computed by the sum of years spent for school career and further training/study.
